# Measuring Agreement in Diagnostics: A Practical Guide for Researchers

**DOI:** 10.1002/sim.70299

**Published:** 2025-10-16

**Authors:** Sophie Vanbelle, Christina Hernandez Engelhart, Ellen Blix

**Affiliations:** ^1^ Methodology and Statistics, CAPHRI Maastricht University Maastricht Limburg the Netherlands; ^2^ Norwegian Research Center for Women's Health Oslo University Hospital Oslo Norway; ^3^ Faculty of Health Sciences Oslo Metropolitan University Oslo Norway

**Keywords:** clinical test, concordance, error, interobserver, intraobserver, observer variation, reliability, repeatability, reproducibility of results

## Abstract

Healthcare professionals routinely perform clinical examinations and diagnostic assessments. How the findings of these assessments are interpreted can have significant implications for patient care and outcomes. A recent systematic review on reliability and agreement studies in intrapartum fetal heart rate monitoring highlighted three methodological issues: (1) confusion between the concepts of agreement and reliability, (2) lack of clarity on how agreement and reliability measures are calculated when more than two raters are involved, and (3) confidence intervals seldom reported. This paper aims to clarify how agreement measures can be computed and interpreted when the outcome is binary (e.g., normal/abnormal test result). Using a motivating example in which five experienced obstetricians assessed 20 CTGs, we demonstrate how agreement can be defined, computed, and interpreted in various scenarios. The paper further explains the relationship between agreement measures and the concept of reliability, the distinction between intra‐ and inter‐observer studies, and approaches to make statistical inference and sample size calculations. Particular emphasis is placed on the proportion of agreement, the proportion of specific agreement and kappa coefficients. A shiny application has also been developed to support researchers in their agreement studies. This work completes existing tools such as the Guidelines for Reporting Reliability and Agreement Studies (GRRAS), the Quality Appraisal Tool for Studies of Diagnostic Reliability (QAREL) and STARD guidelines for reporting diagnostic accuracy studies. It is intended to help researchers improve the methodological quality of studies that evaluate the agreement of clinical tests.

## 1 Introduction

Healthcare professionals perform a wide range of clinical examinations and diagnostic procedures, such as physiological measurements or imaging. The way assessment findings are interpreted has direct consequences for clinical decision‐making and patient outcomes. Variations in interpretation can lead to inconsistencies in care, such as unnecessary interventions or failure to act when necessary. For example, in obstetrics, midwives and obstetricians perform cardiotocography (CTG) to assess fetal well‐being. How the findings are interpreted has direct consequences for the treatment of the mother and her baby.

This underlies the critical importance of the concepts of agreement and reliability in medicine. Agreement is a broad term telling how close clinical assessments are, or to what degree they differ. Reliability refers to the ability of a clinical assessment to distinguish between patients within a particular population. It can be defined as the degree to which the test results can be replicated under different conditions [1].

A recent systematic review [2] on reliability and agreement in intrapartum fetal heart rate monitoring, including 49 studies, pointed out at least three methodological issues when reporting reliability and agreement studies on CTGs classification. In general, information on study design was adequately provided, following guidelines such as GRRAS [3] or STARD [4] although there was considerable heterogeneity in study quality according to QAREL [5]. The agreement measures reported were mainly the proportion of agreement [6], the proportion of specific agreement [7, 8, 9] and (weighted) kappa coefficients [6, 10, 11, 12, 13] while reliability was mainly assessed with kappa coefficients, as advised in the GRRAS guidelines. However, the words agreement and reliability were sometimes used interchangeably although they are two distinct concepts for which statistical measures were independently developed. Agreement measures were mainly developed as descriptive tools on an ad‐hoc basis, whereas reliability measures were grounded in particular mathematical models and assumptions. This confusion could be explained by the fact that study designs for agreement and reliability studies are often similar, and some statistical measures (e.g., kappa coefficients) are presented as both agreement and reliability metrics in the literature (e.g., [11, 14]). For example, in intra‐rater agreement and reliability studies, patients are assessed several times by the same observer (e.g., midwife) under identical conditions. By contrast, in inter‐rater agreement and reliability studies, different observers assess once the same patients under identical conditions.

Furthermore, the computation of agreement and reliability measures was sometimes unclear or not reported, especially in studies involving more than two observers and/or more than two repeated assessments per observer. This is likely because most agreement measures were originally developed for two observers or two repeated assessments and can be extended in various ways. For example, it is straightforward to define agreement between two health professionals: they agree or disagree. With more than two health professionals, we could say that they agree if all agree, if a majority agree, or compute the average agreement between pairs of health professionals, for example.

Finally, confidence intervals were rarely reported, possibly due to the lack of appropriate statistical inference methods and dedicated software. Nonetheless, accounting for statistical uncertainty is essential because we only have information about a sample of patients and observers, while the goal is to draw conclusions about the entire population.

The present paper aims to complement existing guidelines in the context of a binary scale (e.g., normal/abnormal CTG) by clarifying (1) how agreement and reliability measures can be defined in the presence of more than two observers and/or more than two repeated assessments per observer, (2) how to interpret these measures and (3) how statistical inference can be done. A Shiny application (link: https://svanbelle.shinyapps.io/simpleagree/) was developed to facilitate both computations and interpretation of the statistical measures.

The paper is structured as follows. In Section 2, we introduce an example that will illustrate all concepts in this paper. In Section 3, we explain the different assumptions usually made in intra and inter‐observer studies. Section 4 introduces several agreement measures for two observers or two replicates. These statistical measures are extended to more than two observers in Section 5. In Section 6, we interpret kappa coefficients in the context of reliability. In Section 7, we present a method to construct confidence intervals for the measures introduced in Sections 4 and 5. In Section 8, we present how to make sample size calculations when planning a study while in Section 9, we review the possibilities offered in the main statistical software. Finally, we conclude with a discussion in Section 10.

## 2 Case Study: CTG Classification

Consider the hypothetical example presented by Grant [15], which focuses on the agreement level in CTG classification among obstetricians. In this example, five experienced obstetricians (labeled A to E) each assessed 20 cardiotocographs (CTGs), classifying them as either “normal” (negative test result) or “abnormal” (positive test result). Obstetricians A to E rated the following numbers of CTGs as “abnormal”: 6 (30%), 6 (30%), 9 (45%), 7 (35%) and 12 (60%).

The classification can be summarized in a classification table for each pair of obstetricians (see Table 1 for obstetricians A and C).

**TABLE 1:** Classification of 20 CTGs by obstetricians A and C as “normal” or “abnormal” in terms of counts (proportions).

	Obstetrician C		
Obstetrician A	Abnormal	Normal	Total
Abnormal	6 (0.30)	0 (0.00)	6 (0.30)
Normal	3 (0.15)	11 (0.55)	14 (0.70)
Total	9 (0.45)	11 (0.55)	20 (1)

## 3 Difference Between Intra‐ And Inter‐Observer Studies

Two different kinds of agreement (and reliability studies) can be distinguished, namely intra‐ and inter‐observer studies. In intra‐observer studies, replicate assessments of the same patients are made by one observer under identical conditions. The only difference between these replicates is the time at which the assessments are made. In that setting, it is frequently assumed that the order of the assessments does not affect the results, this is known as the *interchangeable ratings assumption* [16]. For instance, if patients are assessed three times by the same observer, the assumption implies that swapping the order of the assessments for some patients will not affect the computed agreement coefficient. Intra‐observer studies are sometimes referred to as repeatability studies [17].

Inter‐observer agreement and reliability studies involve replicate assessments of the same patients conducted by different observers under identical conditions. The interchangeable rating assumption is often not appropriate in inter‐observer studies, since each replicate corresponds to a specific observer with a unique rating style. For example, in the CTG study, obstetrician E classified CTGs as “abnormal” more frequently than the others. It is possible to account for systematic differences in the observers' rating style when the same observers assess all patients. When the set of raters differs between patients, this is not longer possible and the interchangeable ratings assumption should be made [16]. Inter‐observer studies are also known as reproducibility studies [17].

Sometimes, patients are assessed several times by the same set of observers. This design allows for the simultaneous evaluation of both intra‐ and inter‐observer agreement/reliability within a single study. In such cases, the statistical techniques needed to estimate agreement/reliability levels and corresponding confidence intervals become more complex due to the presence of both multiple observers and replicate assessments per observer (e.g., [18]). Nevertheless, the statistical measures introduced in this paper can still be applied by selecting specific assessments. For instance, intra‐observer agreement/reliability can be evaluated separately for each observer. To assess inter‐observer agreement/reliability, one could select specific assessment times for each observer. For example, one can imagine selecting each observer's first assessment on each patient or randomly selecting one assessment per observer. If the interchangeable rating assumption holds within each observer, the method of selection (first or random) should not affect the results.

## 4 Agreement Between Two Observers

Suppose two observers (e.g., midwives) classify N patients or objects (e.g., CTGs) using a binary scale (e.g., “abnormal” and “normal”). Alternatively, a single observer may classify the same N patients at two different assessment times. The assessments can be summarized in a 2×2 classification table, using either counts ($n_{jk}$) or proportions ($p_{jk}$) (see Table 2).

**TABLE 2:** Classification of N patients or objects by two observers using a binary scale (e.g., “abnormal” and “normal”) in terms of counts (proportions).

	Observer 2		
Observer 1	Abnormal (positive test result)	Normal (negative test result)	Total
Abnormal (positive test result)	$n_{11}$ $\left(p_{11}\right)$	$n_{12}$ $\left(p_{12}\right)$	$n_{1.}$ $\left(p_{1.}\right)$
Normal (negative test result)	$n_{21}$ $\left(p_{21}\right)$	$n_{22}$ $\left(p_{22}\right)$	$n_{2.}$ $\left(p_{2.}\right)$
Total	$n_{.1}$ $\left(p_{.1}\right)$	$n_{.2}$ $\left(p_{.2}\right)$	N (1)

*Note:* $n_{11}$: number of objects assessed as abnormal by both observers; $n_{12}$: number of objects assessed as abnormal by observer 1 and normal by observer 2; $n_{21}$: number of objects assessed as normal by observer 1 and abnormal by observer 2; $n_{22}$: number of objects assessed as normal by both observers; $n_{1.}=n_{11}+n_{12}$: number of objects assessed as abnormal by observer 1; $n_{2.}=n_{21}+n_{22}$: number of objects assessed as normal by observer 1; $n_{.1}=n_{11}+n_{21}$: number of objects assessed as abnormal by observer 2; $n_{.2}=n_{12}+n_{22}$: number of objects assessed as normal by observer 2; $N=n_{11}+n_{12}+n_{21}+n_{22}$: total number of objects; $p_{jk}=n_{jk}/N$, j,k=1,2.

### 4.1 Proportion of Agreement

A simple agreement measure is *the proportion of agreement*, denoted by $p_{o}$. It represents the proportion of patients or objects (e.g., CTGs) for which the two observers agree [19, 20]. This measure is also known as the *simple matching coefficient* [21] or the *Rand similarity coefficient* [22],

(1) $$p_{o}=p_{11}+p_{22}.$$

For example, referring to Table 1, obstetricians A and C agree on the classification of 17 CTGs (6 labeled as “abnormal” and 11 as “normal”). This corresponds to a proportion of agreement of $p_{o}=0.30+0.55=0.85$, indicating that obstetricians A and C agree on 85% of the CTG classifications.

### 4.2 Proportion of Specific Agreement

While the proportion of agreement $p_{o}$ provides a simple summary of the overall agreement, it does not differentiate between agreements on positive and negative test results. This can be problematic, especially when the trait under study is relatively rare. In such cases, agreements on negative test results ($p_{22}$) are typically more frequent than those on positive test results ($p_{11}$) and therefore dominate the overall agreement measure $p_{o}$.

To address this limitation, *specific agreement measures* were introduced to focus on agreement within individual categories. For instance, when examining binary outcomes like “abnormal” versus “normal” CTGs, it is often more informative to specifically measure agreement on the “abnormal” cases.

One such measure, *the proportion of positive agreement*, was defined by Dice [7] as

(2) $$p_{pos}=\frac{p_{11}}{\frac{1}{2}(p_{1.}+p_{.1})}.$$

This index ranges from 0 and 1 and can be interpreted as a conditional probability, that is, the probability that an event occurs, given that another event has occurred. In this case, the proportion of positive agreement estimates the probability that both observers agree on positive test results, given the overall probability to classify an object as positive. Similarly, *the proportion of negative agreement* writes

$$p_{neg}=\frac{p_{22}}{\frac{1}{2}(p_{2.}+p_{.2})}.$$

These measures are referred to in the GRRAS guidelines as specific agreement measures [3]. The proportion of positive agreement is also known by various other names, including *Sørensen coefficient* [23], the F‐*measure* or $F_{1}$ *‐score* [24], and the *measure of genetic similarity* [25].

Using the data in Table 1, we have

$$p_{pos}=\frac{0.30}{\frac{1}{2}(0.30+0.45)}=0.80\;\text{and}\;p_{neg}=\frac{0.55}{\frac{1}{2}(0.70+0.55)}=0.88.$$

This indicates that the two obstetricians agree on 80% of the positive test results and 88% of the negative ones, with slightly higher agreement on negative test results.

Goodman and Kruskal [26] suggested the lambda index, λ, for measuring agreement on specific categories, which can be derived from the proportion of positive agreement as $\hat{\lambda }=2p_{pos}-1$. Later, Rogot and Goldberg [27] suggested using the mean of $p_{pos}$ and $p_{neg}$ as an overall agreement index. Another widely used index is the *Jaccard similarity coefficient*, also known as the *Tanimoto coefficient* [28], originally proposed by Jaccard [8] and later by Chamberlain et al. [9]. It is defined as

(3) $$p_{J}=\frac{p_{11}}{p_{11}+p_{12}+p_{21}}=\frac{p_{11}}{p_{1.}+p_{.1}-p_{11}}.$$

Like $p_{pos}$, the Jaccard similarity coefficient takes values between 0 and 1 and can be interpreted as a conditional probability. It gives the proportion of agreement on positive test results given that either of the observers rates a patient as positive.

The careful reader would have remarked the similarity between Equations (2) and (3). In fact, there is a direct relationship between the two coefficients given by $p_{J}=p_{pos}/(2-p_{pos})$ and $p_{pos}=2p_{J}/(1+p_{J})$. Thus, the choice between the two coefficients is largely one of interpretative preference. Note that Grant [15] referred to the Jaccard coefficient as “*the proportion of agreement*”, which can lead to confusion with $p_{o}$.

Considering Table 1, Jaccard similarity coefficient is equal to $p_{J}=0.30/(0.30+0.00+0.15)=0.67$. Therefore, given an “abnormal” rating by either obstetrician, the proportion of agreement on the positive test results is 0.67. Notably, if $p_{J}\leq 0.5$, it implies that, given a positive rating from either observer, the second observer is not doing better than what would be expected by flipping a fair coin [15].

Finally, there is a mathematical relationship linking $p_{o}$, $p_{pos}$ and $p_{neg}$. The proportion of agreement $p_{o}$ is a weighted average of the proportions of specific agreement,

$$p_{o}=\left(\frac{p_{1.}+p_{.1}}{2}\right)p_{pos}+\left(\frac{p_{2.}+p_{.2}}{2}\right)p_{neg}.$$

Here, $p_{pos}$ is weighted by the overall proportion of positive test results and $p_{neg}$ by that of negative test results, respectively. Consequently, when positive test results are rare, $p_{o}$ will be dominated by the proportion of negative agreement $p_{neg}$.

### 4.3 Chance‐Corrected Agreement

The above agreement indexes were criticized because some agreement may occur purely by “chance”. That is, even when observers classify the patients randomly, they will agree to some extent. To address this, kappa coefficients (κ) were introduced. These compare the proportion of agreement $p_{o}$ to some proportion of agreement expected by chance. The general form of the kappa statistic is

(4) $$\hat{\kappa }=\frac{p_{o}-p_{e}}{1-p_{e}}=1-\frac{q_{o}}{q_{e}}$$

where $p_{o}$ is the proportion of agreement defined in Equation (1), $q_{o}=1-p_{o}$ is the proportion of disagreement, $p_{e}$ is the proportion of agreement expected by chance and $q_{e}=1-p_{e}$ is the proportion of disagreement expected by chance. A value of 1 indicates perfect agreement, a value of 0 indicates agreement not better than chance, and negative values suggest worst than chance agreement.

Over time, several definitions of chance were introduced (e.g., [10, 11, 12, 29, 30]). Three commonly used definitions of chance agreement, possessing a straightforward interpretation and related to each other are given below.

Chance agreement between two observers is defined by imagining that each observer classifies patients or objects by tossing a coin.

Def1 Completely random classification. Each observer uses a fair coin, assigning categories with equal probability (0.5). This leads to $p_{e}=0.5\times 0.5+0.5\times 0.5=0.5$ and $q_{e}=0.5$.
Def2 Observer‐specific marginal classification. Each observer uses an unfair coin, with a probability of getting heads equal to the proportion of patients or objects classified as positive test results by the observer (i.e., with marginal probability estimated by $p_{1.}$ and $p_{.1}$). This definition accounts for possible differences in the rating style of the observers. This leads to $p_{e}=p_{1.}p_{.1}+p_{2.}p_{.2}$ and $q_{e}=1-p_{1.}p_{.1}-p_{2.}p_{.2}$.
Def3 Average marginal probabilities. Observers are assumed to use the same unfair coin, with a probability of getting heads equal to the overall proportion of positive test results. This definition assumes that the two observers have the same rating style. This leads to $p_{e}={\left(\frac{p_{1.}+p_{.1}}{2}\right)}^{2}+{\left(\frac{p_{2.}+p_{.2}}{2}\right)}^{2}$ and $q_{e}=1-{\left(\frac{p_{1.}+p_{.1}}{2}\right)}^{2}-{\left(\frac{p_{2.}+p_{.2}}{2}\right)}^{2}=2\left(\frac{p_{1.}+p_{.1}}{2}\right)\left(\frac{p_{2.}+p_{.2}}{2}\right)$.

Each of these chance definitions corresponds to a different version of the kappa statistic. Using definition 1, the resulting kappa is known as the *G index* [19], *PABAK* [31], *Brennan and Prediger kappa* or the *free marginal kappa* [29]. Using definition 2, it is known as *Cohen's kappa coefficient* [11] and using definition 3 as *Scott's pi* [10] or the *intraclass kappa coefficient* [14]. Note that using chance definition 1, the kappa coefficient is a simple function of $p_{o}$, that is, ${\hat{\kappa }}_{1}=2p_{o}-1$.

We have, for the example in Table 1,

Def1 $p_{e}=0.5\times 0.5+0.5\times 0.5=0.50$, leading to ${\hat{\kappa }}_{1}=\frac{0.85-0.50}{1-0.50}=1-\frac{0.15}{0.50}=1-0.30=0.70$.
Def2 $p_{e}=0.30\times 0.45+0.70\times 0.55=0.52$, leading to ${\hat{\kappa }}_{2}=\frac{0.85-0.52}{1-0.52}=1-\frac{0.15}{0.48}=1-0.31=0.69$.
Def3 $p_{e}={\left(\frac{0.30+0.45}{2}\right)}^{2}+{\left(\frac{0.70+0.55}{2}\right)}^{2}=0.53$, leading to ${\hat{\kappa }}_{3}=\frac{0.85-0.53}{1-0.53}=1-\frac{0.15}{0.47}=1-0.32=0.68$.

Kappa coefficients are often more intuitive when interpreted in terms of disagreements than in terms of agreements. For example, under chance definition 2 (Cohen's kappa), we have ${\hat{\kappa }}_{2}=1-0.15/0.48=0.69$. This means that the proportion of disagreement ($q_{o}=0.15$) is 0.31 times (=1−0.69) the proportion of disagreement expected by chance, accounting for the observers' rating style.

In the example, all three kappa values are close, but slightly different due to how chance is defined. In general, the following inequality holds, ${\hat{\kappa }}_{1}$ ≥ ${\hat{\kappa }}_{2}$ ≥ ${\hat{\kappa }}_{3}$. we have ${\hat{\kappa }}_{2}={\hat{\kappa }}_{3}$ when both observers classify the same proportion of patients or objects as positive. If this common proportion equals 0.5, then all three kappa coefficients will coincide. Therefore, a difference between ${\hat{\kappa }}_{2}$ and ${\hat{\kappa }}_{3}$ indicates that the proportion of positive test results differs between the two observers. If ${\hat{\kappa }}_{2}>{\hat{\kappa }}_{3}$ and ${\hat{\kappa }}_{2}>{\hat{\kappa }}_{1}$, it suggests a large difference between $p_{1.}$ and $p_{.1}$, with one of the two proportions exceeding 0.5 and the other below 0.5 [32, 33].

Several alternative measures have also been proposed. For example, Krippendorf alpha [34] also builds on chance definition 3, but incorporates Bessel's correction to adjust for finite sample sizes. Specifically, the chance disagreement is modified as

$$q_{e}=2\left(\frac{NR-1}{NR}\right)\left(\frac{p_{1.}+p_{.1}}{2}\right)\left(\frac{p_{2.}+p_{.2}}{2}\right).$$

The resulting $q_{e}$ can therefore be interpreted as chance disagreement when sampling from the patients population without replacement. This reasoning is usually made when sampling from finite populations of patients.

## 5 Agreement Between More Than Two Observers

Suppose now that N patients or objects are assessed by the same set of R observers (inter‐rater agreement), as in the example of Grant [15], or that a single observer assesses the same N patients on R occasions (intra‐rater agreement). In both situations, we obtain R assessments per patient.

While agreement is easy to define between two observers or more generally between two replicate assessments (either they agree or disagree), this is not the case for more than two replicates. For instance, in inter‐rater agreement studies, one might define agreement as requiring all R observers to agree, or alternatively, agreement could be based on a majority consensus. However, the most common approach is to compute a weighted mean over all observer pairs perhaps for two reasons. Agreement coefficients were primarily defined between two observers and it is difficult to decide on a majority rule. Notably, all agreement coefficients presented in this section reduce to their two‐observer counterpart presented in Section 4 when R=2.

### 5.1 Proportion of Agreement

For multiple observers, the most common generalization of the proportion of agreement $p_{o}$ is the mean pairwise proportion of agreement, the average agreement over all R(R−1)/2 distinct observer pairs [13, 35],

(5) $$\begin{array}{cc} p_{o} & =\text{mean over all pairs of}\;p_{o}^{\left(p\right)}\\ & =\frac{1}{NR(R-1)}\overset{N}{\underset{i=1}{\sum }}[m_{i1}(m_{i1}-1)+m_{i2}(m_{i2}-1)] \end{array}$$

where the superscript (p) denotes a pair p=1,⋯,R(R−1)/2, $m_{ij}$ is the number of observers assigning patient i (i=1,⋯,N) to category j (j=1,2) and $m_{i2}=R-m_{i1}$. The term $m_{ij}(m_{ij}-1)/2$ gives the number of agreeing observer pairs for patient i and category j. If the number of observers varies per patient and is equal to $R_{i}$ for patient i, the formula becomes

$$p_{o}=\frac{1}{N}\overset{N}{\underset{i=1}{\sum }}\frac{1}{R_{i}(R_{i}-1)}[m_{i1}(m_{i1}-1)+m_{i2}(m_{i2}-1)]$$

where $m_{i2}=R_{i}-m_{i1}$.

In the obstetric example, the mean proportion of agreement across all obstetricians is $p_{o}=0.73$, meaning that, on average, any pair of obstetricians agrees on 73% of the CTG classifications. To gain more insight into the pattern of disagreement, the proportion of agreement is reported in Table 3 for all distinct pairs of obstetricians.

**TABLE 3:** Obstetrical example. Proportion of agreement for all distinct pairs of obstetricians.

	B	C	D	E
A	0.90	0.85	0.85	0.60
B		0.85	0.95	0.50
C			0.90	0.45
D				0.45

*Note:* For example, the number in the cell (A,B) represents the proportion of agreement between obstetricians A and B.

It can be observed in Table 3 that the proportion of agreement ranges from 0.45 to 0.90 and is lower when obstetrician E is involved. This is because obstetrician E rates CTGs as “abnormal” nearly twice as often as the others. When excluding obstetrician E, pairs of observers agree on average on 88% of the CTGs.

### 5.2 Proportion of Specific Agreement

Specific agreement coefficients can also be generalized in many ways. For example, specific agreement between many observers can be obtained (1) by computing the index for all observer pairs and take the mean or (2) by computing the mean numerator and mean denominator separately across all pairs and take the ratio. The first approach offers straightforward interpretation. It is the mean specific agreement over all pairs of observers. In the example of Grant [15], this leads to $p_{pos}=0.69$ and $p_{J}=0.56$. However, this approach lacks desirable mathematical properties. For instance, the relationship $p_{J}=p_{pos}/(2-p_{pos})$ (see Section 4), not longer holds, and analytical formula cannot be derived to compute confidence intervals (see Section 7). Thus, the second generalization is generally preferred [15, 36]. This gives

(6) $$p_{pos}=\frac{\text{mean over all pairs of}\;p_{11}^{\left(p\right)}}{\text{mean over all pairs of}\;\frac{\left(p_{1.}^{\left(p\right)}+p_{.1}^{\left(p\right)}\right)}{2}}=\frac{1}{R-1}\left(\frac{\sum_{i=1}^{N}m_{i1}^{2}}{\sum_{i=1}^{N}m_{i1}}-1\right).$$

where the superscript (p) denotes the pair p=1,⋯,R(R−1)/2. The proportion of negative agreement and Jaccard index can be generalized similarly.

This method preserves key mathematical relationships (e.g., $p_{J}=p_{pos}/(2-p_{pos})$) and allows analytical formulas for confidence intervals. However, it introduces weighting, favoring observer pairs with more frequent positive classifications. As such, the result reflects an average that accounts for each pair's classification profile. The specific agreement indexes derived under approaches 1 and 2 are equal only when the proportion of positive test results is the same in all pairs of observers.

In the obstetric example, when considering the mean of the indexes between all possible pairs (approach 1), we have $p_{pos}=0.69$ and $p_{J}=0.56$. Under the second approach, we obtain $p_{pos}=0.66$ and $p_{J}=0.50$. The difference in the results of the two approaches shows some discrepancies in the proportion of positive test results between pairs. By excluding obstetrician E, who rates CTGs as “abnormal” more often than the other obstetricians, we obtain under the first approach $p_{pos}=0.83$ and $p_{J}=0.71$ and under the second approach $p_{pos}=0.83$ and $p_{J}=0.72$. The two approaches lead to similar coefficients, reflecting the higher homogeneity between observers in the proportion of “abnormal” CTGs.

### 5.3 Chance‐Corrected Agreement

Chance‐corrected agreement coefficients were also extended to more than two observers under the second approach (i.e., weighted mean over all distinct pairs of observers) and are known as *kappa‐q, kappa‐BP* [30], or R*andolph's kappa* [37] (chance definition 1), *Hubert's kappa* [38] or *Conger kappa* [13, 35] (chance definition 2) and *Fleiss kappa* [39] (chance definition 3). It is therefore important to note that Fleiss kappa is not the generalization to more than two observers of Cohen's kappa but of Scott's pi.

The general formula is [13, 35]

(7) $$\begin{array}{cc} \hat{\kappa } & =\frac{p_{o}-p_{e}}{1-p_{e}}\\ & =\frac{\text{mean over all pairs of}\;(p_{o}^{\left(p\right)}-p_{e}^{\left(p\right)})}{\text{mean over all pairs of}\;(1-p_{e}^{\left(p\right)})}\\ & =1-\frac{\text{mean over all pairs of}\;q_{o}^{\left(p\right)}}{\text{mean over all pairs of}\;q_{e}^{\left(p\right)}}. \end{array}$$

where $p_{o}$ is given by Equation (5). The proportion of expected agreement $p_{e}$ depends on the chosen chance definition. Under chance definition 1 (completely random classification), $p_{e}=0.5$. Under chance definition 2 (observer‐specific marginal classification), we have

(8) $$p_{e}=\overset{2}{\underset{j=1}{\sum }}p_{j}^{2}-\frac{1}{R(R-1)}\overset{2}{\underset{j=1}{\sum }}\overset{R}{\underset{r=1}{\sum }}{\left(p_{j(r)}-p_{j}\right)}^{2}$$

where $p_{j}=\sum_{i=1}^{N}m_{ij}/(NR)$ is the overall proportion of patients or objects classified in category j (j=1,2) and $p_{j(r)}$ is the proportion of patients or objects that observer r classified in category j (j=1,2;r=1,⋯,R). Under chance definition 3 (average marginal probabilities), we have

(9) $$p_{e}=\frac{1}{N}\overset{N}{\underset{i=1}{\sum }}\frac{1}{R}(m_{i1}p_{1}+m_{i2}p_{2}).$$

Note that if the number of observers differs between patients or objects, we have

$$p_{e}=\frac{1}{N}\overset{N}{\underset{i=1}{\sum }}\frac{1}{R_{i}}(m_{i1}p_{1}+m_{i2}p_{2})$$

under chance definition 3, with $p_{j}=\sum_{i=1}^{N}m_{ij}/(NR_{i})$.

In the CTG example, we have ${\hat{\kappa }}_{1}=0.66$ under chance definition 1, ${\hat{\kappa }}_{2}=0.45$ under definition 2, and ${\hat{\kappa }}_{3}=0.44$ under definition 3. By excluding obstetrician E, we obtain ${\hat{\kappa }}_{1}=0.76$ (definition 1), ${\hat{\kappa }}_{2}=0.75$ (definition 2) and ${\hat{\kappa }}_{3}=0.74$ (definition 3). Let us interpret the coefficient obtained under chance definition 2 to account for possible differences in the observers' rating style. On average, the proportion of disagreement (17% = $1-p_{o}$) is about 0.55 times the proportion of disagreement expected by chance ($1-{\hat{\kappa }}_{2}$ = 1‐0.45 = 0.55). Excluding obstetrician E, the proportion of disagreement drops to 12% (= $1-p_{o}$), about a quarter of the proportion of disagreement expected by chance.

## 6 Kappa Coefficients in the Context of Reliability Studies

Reliability refers to the ability of a measurement instrument to distinguish between patients or objects in a population. While originally developed for continuous scales, the concept has since been extended to binary scales, although this introduces additional challenges.

The idea of reliability stems from classical test theory, which models any observed score as the sum of a true score and measurement error [40]. In that framework, reliability was first defined as the squared correlation between the observed and true scores [41]. Because true scores are usually unobservable, researchers estimate reliability using replicate measurements. When the replicates are assumed to share the same true score and error structure, the correlation between them is known as the *intraclass correlation coefficient (ICC)*. The ICC ranges from 0 to 1. A value close to 1 indicates that most variability in the measurements is due to true differences between patients or objects, suggesting high reliability. In contrast, values near 0 suggest that measurement error dominates. Importantly, reliability depends on population homogeneity. It can be low in homogeneous populations because a same amount of measurement error will appear more important when compared to the variability between patients in homogeneous populations than in heterogeneous populations.

As study designs became more complex, classical test theory was extended. Generalizability theory defines reliability as a variance ratio, accounting for multiple sources of variation such as observer effects and measurement error [42] while, the correlation‐based approach generalizes ICCs by assuming more complex models for the replicates [43, 44]. For the study designs considered in this paper (intra‐ and inter‐rater designs), these approaches lead to the same reliability coefficients.

Extending reliability to binary outcomes (e.g., normal vs. abnormal) is difficult because of differences in how measurement error behaves. For continuous scales, measurement error refers to random fluctuations around a continuous true score. For binary scales, measurement error refers to a misclassification probability, that is, assigning a patient to the wrong category. Since the true category is usually unknown, replicate assessments are used to estimate the probability of being classified in each category. This leads to a key challenge: while the observed binary value is 0 or 1, the true underlying value is better interpreted as a probability, a continuous concept. Bridging this gap requires latent variable models, such as item response theory, probit, or logit regression, which link the binary observed outcome to an underlying continuous latent trait.

These models allow for two types of reliability: latent and manifest scale reliability. At the latent scale level, reliability aligns with the continuous definition. It reflects the proportion of latent score variance attributable to the true latent trait rather than measurement error [45]. Manifest scale reliability, which attempts to quantify reliability at the binary outcome level, is more problematic since the variance of a binary variable depends on the outcome prevalence, not just measurement accuracy.

To estimate reliability at the manifest scale level, several approaches have been proposed. The normal approximation approach treats the binary scale as a continuous scale and mimics the continuous case [45, 46, 47]. While easy to use, this approach ignores the binary nature of the data, which is problematic when constructing confidence intervals [48]. In the latent variable approach, several methods were developed to approximate the manifest scale reliability using information at the latent scale level [45, 49, 50, 51]. These methods better respect the binary nature of the scale.

Although originally introduced as agreement measures, kappa coefficients under chance definitions 2 and 3 were shown to be very close to the ICCs defined under the normal approximation approach, especially when the number of patients or objects exceeds 20 [11, 13, 14, 52]. The kappa coefficient under chance definition 3 (${\hat{\kappa }}_{3}$) aligns with classical test theory, where observed scores are decomposed into a true score and random error, that is, under a one‐way ANOVA model. On the other hand, the kappa coefficient under chance definition 2 (${\hat{\kappa }}_{2}$), accounting for possible differences in the rating style of the observers, is very close to the ICC obtained when an observed score is decomposed as a true score, an observer effect and measurement error, that is, under a two‐way ANOVA model.

As such, ${\hat{\kappa }}_{2}$ is often advised in inter‐rater reliability studies while ${\hat{\kappa }}_{3}$ is more often used in intra‐rater reliability studies. One advantage of using kappa coefficients over applying the normal approximation approach directly is that the binary character of the scale is taken into account when constructing confidence intervals. This results in better statistical properties [48]. Further, these two kappa coefficients were also shown to be very close to the manifest scale reliability obtained in the latent variable approach [14, 48]. In summary, kappa coefficients can be interpreted as reliability coefficients for binary scales when sample sizes are large. This also explains why, like ICCs, kappa coefficients are influenced by the population homogeneity. Kappa coefficients can be close to 0 (e.g., ${\hat{\kappa }}_{3}∼0$) despite a good proportion of agreement (e.g., $p_{o}=0.85$), especially in homogeneous populations [33].

In the motivating example, using chance definition 2, we have ${\hat{\kappa }}_{2}=0.45$. This means that about 45% of the variability between the CTGs classifications can be explained by the variability between the patients on which the CTGs were made, that is, about 55% of the variability is due to other sources including differences between the obstetricians and measurement error. Caution should be taken when interpreting these values as such because this interpretation is made possible by treating the binary scale as a continuous scale (see [48] for further clarification and discussion).

## 7 Statistical Inference

It is important to report confidence intervals with agreement coefficients, as they reflect the uncertainty associated with estimating agreement from a sample of observers and patients. When the number of observers or patients is too small, confidence intervals can become so wide that they fail to provide useful conclusions. To avoid such situations, sample size calculations can be performed during the planning phase of the study (see Section 8). Before discussing sample size considerations, however, we first explain how to construct confidence intervals for agreement coefficients.

Although several frameworks are available for inference (e.g., Bayesian, frequentist), we focus on a specific frequentist approach, as it is the one most commonly implemented in statistical software.

A straightforward and widely applicable method for constructing confidence intervals is the (1−α)% Wald confidence interval, given by

(10) $$\hat{measure}\pm Q_{z}(1-\alpha /2)SE(\hat{measure})$$

where $\hat{measure}$ is one of the statistical measures presented in Sections 4 and 5 evaluated on a sample of patients or objects, $Q_{z}(1-\alpha /2)$ is the 100(1−α/2) percentile of the standard normal distribution (e.g., the 95% percentile is equal to $Q_{z}(0.975)=1.96$) and $SE(\hat{measure})$ is the standard error of the statistical measure.

There are several methods to determine the standard error of agreement/reliability coefficients, in particular kappa coefficients, because there is no formula possessing good statistical properties across the full range of kappa values (0 to 1).

In this paper, we use the delta method (see Appendix A for formulas) for two reasons. First, this generic method applies to all coefficients presented in this paper. Second, it generally produces confidence intervals with good statistical properties, except when the number of patients and/or observers is small (e.g., two observers and less than 30 patients) or when agreement/reliability levels are very high (e.g., >0.90) [53, 54, 55]. It is difficult to obtain confidence intervals with good statistical properties whatever the method used with small sample sizes because the distribution of agreement/reliability levels is discrete. For example, with three observers and one patient, the proportion of agreement can only take values 0/3, 1/3, 2/3, and 3/3, that is, not any value between 0 and 1. This phenomenon is known to affect the statistical properties of confidence intervals for proportions [56]. To mitigate this issue in the special case of two observers, Yates' continuity correction can be applied to improve the confidence interval for the proportion of agreement (see Appendix A). When agreement levels are close to one (e.g., >0.90), the Wald method may also perform poorly because it assumes a normal distribution for the agreement/reliability coefficients, whereas agreement coefficients are bounded by 1, which induces a skewed distribution. In such cases, alternative methods such as non‐parametric bootstrap confidence intervals [54] or Fisher Z‐transformation [48, 53] may offer better performance.

For the CTG example, confidence intervals for the various agreement/reliability coefficients are given in Table 4 for observers A and C, observers A to E and observers A to D.

**TABLE 4:** Obstetrical example.

	$p_{o}$	${\hat{\kappa }}_{1}$ (def 1)	${\hat{\kappa }}_{2}$ (def 2)	${\hat{\kappa }}_{3}$ (def 3)	$p_{pos}$	$p_{neg}$
**Observers A and C**						
Estimate	0.85	0.70	0.69	0.68	0.80	0.88
Lower bound	0.69	0.39	0.38	0.35	0.58	0.75
Upper bound	1.00	1.00	1.00	1.00	1.00	1.00
**Observers A to E**						
Estimate	0.73	0.46	0.45	0.44	0.66	0.78
Lower bound	0.63	0.26	0.22	0.20	0.47	0.68
Upper bound	0.83	0.66	0.67	0.67	0.86	0.87
**Observers A to D**						
Estimate	0.88	0.77	0.75	0.74	0.83	0.91
Lower bound	0.78	0.56	0.52	0.52	0.67	0.82
Upper bound	0.99	0.97	0.97	0.97	0.99	1.00

*Note:* 95% Wald confidence intervals (lower bound, upper bound) with standard error derived by the delta method for the proportion of agreement ($p_{o}$), kappa coefficients (${\hat{\kappa }}_{1}$, ${\hat{\kappa }}_{2}$, ${\hat{\kappa }}_{3}$), the proportion of positive agreement ($p_{pos}$) and the proportion of negative agreement ($p_{neg}$).

From Table 4, we observe that even when kappa coefficients are around 0.45 (e.g., for observers A to E), the 95% confidence interval ranges approximately from 0.20 to 0.70, covering about half of the possible values. This indicates a high level of uncertainty about the population‐level agreement. Similarly, the confidence interval for the proportion of positive agreement ranges from 0.47 to 0.86, yielding a width of 0.40 on a 0‐1 scale. This underlines the importance of reflecting the uncertainty associated with the sampling process, as we only have an imperfect view of the reality when considering a sample.

To reduce the width of confidence intervals, studies should include a larger number of patients and/or observers during the planning phase (see Section 8).

## 8 Sample Size Calculation

When planning an agreement study, it is important to determine the number of observers and patients or objects that will be included. There are two common approaches to guide this planning: (1) estimating agreement/reliability with a desired level of precision (i.e., a targeted confidence interval width) or (2) testing statistical hypotheses about agreement/reliability. In both cases, the most common situation is to determine the minimum number of patients needed for a given number of observers. The formulas derived in this section are based on that assumption. Furthermore, since it is generally difficult to anticipate how the tendency to give a positive test result may vary across observers, it is often assumed that this proportion is the same for all observers.

Under these two assumptions, the minimum information needed to determine the minimum number of observers and patients or objects required includes the expected proportion of positive test results and the target agreement level. For some agreement measures, additional information, often difficult to determine in advance, is also required [53]. For instance, estimating the proportion of positive agreement necessitates to make assumptions about expected agreement levels among triplets of observers.

### 8.1 Confidence Interval Approach

In the confidence interval approach, we would like to achieve a confidence interval with a width smaller than a predefined value w around the agreement level. Using Wald confidence interval, the width is given by $2Q_{z}(1-\alpha /2)SE(\hat{measure})$ (see Equation (10)) where the form of $SE(\hat{measure})$ depends on the agreement measure considered. To determine the minimum number of patients or objects (N) needed to achieve the desired precision, we solve the above equation for N. Since an analytical solution is not always available, numerical procedures are used (see Appendix B for details specific to the agreement coefficients considered in this paper).

Consider the study of Grant [15] as a pilot study for planning purposes. Researchers are interested in estimating the proportion of agreement, expected to be around 0.80, and wish to achieve a 95% confidence interval width smaller than 0.10, that is, a 95% confidence interval (0.75,0.85). Assume that they can recruit between 3 and 8 obstetricians, and based on clinical experience, the expected proportion of “abnormal” cases is approximately 0.35. Using the numerical evaluation procedure described in Appendix B, the minimum number of patients needed to meet the confidence interval criterion is given in Table 5.

**TABLE 5:** Minimum number of patients needed according to the number of observers to achieve a proportion of agreement of 0.80 with a 95% confidence interval width of 0.10 or less assuming 35% of “abnormal” cases.

Number of observers	3	4	5	6	7	8
Minimum number of patients	144	144	121	111	106	95

To determine the most realistic combination of the number of observers and patients, one must also consider practical constraints, such as potential observer withdrawals, missing data and the time burden to review CTGs. If interpreting one CTG takes approximately 5 min, each obstetrician should ideally assess no more than 120 CTGs. Based on this constraint, a feasible study design would include 6 observers and 120 CTGs.

### 8.2 Testing Approach

In the testing approach, the objective is to test statistical hypotheses of the form

$$H_{0}:\rho \leq \rho_{0}\;\text{versus}\;H_{1}:\rho >\rho_{0},\;\text{that is,}\rho =\rho_{A},$$

where $\rho_{0}$ is the agreement level under the null hypothesis, $\rho_{A}$ under the alternative hypothesis, 1−β the pre‐specified power and α the type‐one error. The minimum number of patients, N, required to test this hypothesis is obtained by solving the following equation for N,

$$\Phi_{z}\left(\frac{\rho_{A}-\rho_{0}}{\sqrt{var(\rho_{A})}}-Q_{z}(1-\alpha )\right)\geq 1-\beta$$

where Φ(.) is the cumulative distribution function of the standard normal distribution and $\sqrt{var(\rho_{A})}$ is the standard error of the agreement coefficient under the alternative hypothesis. As with the confidence interval approach, this equation typically cannot be solved analytically, and numerical methods are used (see Appendix B).

Again, take the study by Grant [15] as a basis. Researchers want to test the above hypotheses for the proportion of agreement with $\rho_{0}=0.80$, $\rho_{A}=0.85$, α=0.05 and 1−β=0.80. Again, they can recruit between 3 and 8 obstetricians and the proportion of “abnormal” cases is expected to be around 0.35. The minimal number of patients needed to achieve the desired power is given in Table 5.

Taking into account the same constraints discussed earlier, we could plan a study with 6 observers and 110 CTGs in the context of the testing approach (Table 6).

**TABLE 6:** Minimum number of patients needed according to the number of observers to test the statistical hypotheses about the proportion of agreement with $\rho_{0}=0.80$, $\rho_{A}=0.85$, α=0.05 and 1−β=0.80 and an expected proportion of “abnormal” cases of 0.35.

Number of observers	3	4	5	6	7	8
Minimum number of patients	191	138	111	91	78	68

## 9 Statistical Software

The systematic use of certain agreement coefficients, may, in part, be explained by the limited options offered in statistical software. Most statistical packages allow the computation of Cohen's kappa (i.e., ${\hat{\kappa }}_{2}$) for two observers and Fleiss kappa (i.e., ${\hat{\kappa }}_{3}$) for more than two observers. However, in many cases, the standard errors and confidence intervals provided by these software packages are not of general form. Specifically, the standard error is often calculated under the null hypothesis that κ=0, which tends to produce confidence intervals that are too narrow and thus unreliable for general inference (see Table 7). The proportion of agreement ($p_{o}$) and specific agreement can typically only be obtained for two observers. Even then, standard errors are not provided by commonly used packages such as SPSS, STATA, and SAS. In contrast, the R package irrCAC, allows users to compute $p_{o}$ for two or more observers, along with a 95% confidence interval.

**TABLE 7:** Statistical software.

Statistical software	SPSS	STATA	SAS	R
${\hat{\kappa }}_{2}$ (2 observers)	crosstab^a^	kap^a^, ^b^, kapci^c^	PROC FREQ^a^	irr^a^, ^b^
${\hat{\kappa }}_{2}$ (2 or more observers)				irrCAC^d^
${\hat{\kappa }}_{3}$ (2 or more observers)	reliability	kappa^a^, ^b^	MAGREE^a^, ^b^	irr^a^, ^b^
	analysis^a^, ^b^	kappac^c^	INTER_RATER^a^	irrCAC^d^
$p_{o}$, $p_{pos}$, $p_{J}$ (2 observers)		matrix	PROC DISTANCE^e^	catsim^e^
		dissimilarity^e^		
$p_{o}$ (2 or more observers)				irrCAC^d^

*Note:* Procedures for computing agreement measures and methods for constructing confidence intervals.

^a^ Wald confidence interval and delta method.

^b^ The standard error is not valid in general but only to test the statistical hypothesis κ=0.

^c^ Bootstrap confidence interval.

^d^ Based on a linearization technique, very close to Wald confidence interval and delta method.

^e^ No standard error provided.

When it comes to sample size calculation, the limitations of existing software become even more pronounced. For two observers and the kappa coefficient defined under chance definition 2 (Cohen's kappa), STATA provides the commands sskapp and kapssi, which can estimate the minimum number of patients required to achieve a specified confidence interval width around the expected agreement level. Similarly, the R package vcd provides tools for hypothesis testing in this context. For chance definition 3 and two observers or more (Fleiss' kappa), the sskapp command in STATA also supports sample size estimation. The R package kappasize provides similar functionality but is currently limited to six observers.

To address these limitations, we developed a shiny app and an accompanying R package to assist researchers in selecting the most appropriate statistical measures, constructing confidence intervals and performing sample size calculations. This shiny application does not require programming skills, making it accessible to a broad range of users. A step by step procedure to perform the statistical analyses presented in this paper is provided as Supporting Information.

## 10 Discussion

This paper reviewed the agreement measures most commonly used in medical research for binary scales, with particular emphasis on clarifying how these coefficients should be interpreted. Like several other authors, we do not advise the use of standard tables such as Landis and Koch sale [57] to label the strength of agreement [57] (e.g., poor, fair, substantial) for at least three reasons. First, such classification is subjective. The interpretation of agreement levels should be adapted to the context in which the measurement instrument is used. For example, an agreement level of 0.70 may be acceptable when measuring heart rate among recreational runners but would likely be insufficient in a medical context. Moreover, in psychology, where constructs like intelligence are complex and difficult to evaluate, researchers could be satisfied with lower agreement levels than in some medical context where more objective measurements are made (e.g., measuring the ankle of a knee). Second, despite being developed for a specific kappa coefficient, the Landis and Koch scale [57] is often misapplied to other statistical measures. As illustrated in Table 4, we see that agreement can be qualified as “moderate” or “substantial”, depending on the statistical measure considered. Third, these classifications typically ignore statistical uncertainty, that is, confidence intervals. For example, an observed agreement level of 0.71 might be labeled as “substantial,” with a lower bound of the confidence interval being 0.69 or 0.49.

Different agreement coefficients capture different aspects of (dis)agreement patterns, and each has its strengths and limitations. For this reason, it is advisable to report multiple coefficients rather than relying on a single statistic. The proportion of agreement is simple to interpret but mixes agreement on the two categories. As such, it could not provide enough insight when, for example, the trait under study is rare. Specific agreement coefficients provide a more granular view by separating agreement obtained on each category. However, neither of these measures account for agreement expected by chance. Over the years, several kappa coefficients have been proposed, each based on a distinct definition of chance agreement. In this paper, we reviewed three chance definitions. Chance definition 1 is rarely used in practice because the resulting kappa is a simple linear transformation of the proportion of agreement, this latter being easier to interpret. In general, when the same observers classify the same patients on a binary scale, chance definition 2 is advised, as it takes into account the rating style of the observers. Chance definition 3 assumes that all observers have the same probability of giving a positive result and is advised in intra‐rater agreement studies or when different set of raters assess each patient. Krippendorf alpha generally yields values similar to Fleiss kappa coefficient since the expected disagreement under chance is only slightly different between the two. Another popular alternative to kappa coefficients is Gwet's AC1 [58], expressible as ${\hat{AC}}_{1}=(p_{o}-q_{e})/(1-q_{e})$ where $q_{e}$ is the proportion of disagreement expected by chance under definition 3. However, this formulation compares the observed agreement to the expected disagreement, which has been criticized as conceptually misleading [59].

It is important to explicitly state the definition of chance agreement used, as the value of the kappa coefficient is directly affected by it. Further, under chance definitions 2 and 3, the kappa coefficients are closely related to reliability coefficients derived from two‐way and one‐way ANOVA models, respectively. These models treat the binary outcome as continuous. This explains why kappa coefficients are sometimes referred to as reliability measures. The concept of reliability denotes the extent to which a measurement instrument can distinguish between patients in a specific population. These two kappa coefficients therefore depend on the homogeneity of the population (i.e., the number of positive and negative test results). In highly homogeneous populations, where most test outcomes are similar, it becomes inherently difficult to differentiate between patients. As a result, kappa values can appear low even when the proportion of observed agreement is high [33]. It is therefore essential to report the proportion of positive test results for each observer, as this information helps to contextualize unexpectedly low kappa values despite a high proportion of agreement. Further, for reproducibility, authors should document exactly how agreement and reliability coefficients were computed, especially in settings involving more than two observers. In such cases, multiple definitions of agreement (e.g., consensus, pairwise) and multiple statistical measures based on different assumptions (e.g., different definitions of chance agreement) exist. While reliability is commonly quantified using kappa coefficients in the literature, a more in‐depth discussion on measurement error and reliability in the context of binary scales is needed (see e.g., [48]). Unlike continuous scales, binary classifications offer limited variability, raising the question of whether definitions of reliability for continuous scales remain appropriate.

Beyond interpretation, sample size planning is another critical but often overlooked aspect of agreement studies. A too small sample size may produce confidence intervals so wide that the resulting conclusions become practically meaningless. Note that in some situations (e.g., proportion of positive agreement with more than two observers), it is hardly possible to have a single number for the minimum sample size [53]. Nevertheless, some idea about the magnitude of the minimum sample size can be obtained. Moreover, including more than two observers is generally recommended, not only for statistical purposes but also for generalizability purposes. In addition, other aspects of study design should not be overlooked. For instance, in intra‐rater studies, the time interval between repeated assessments is critical: it must be sufficiently long to reduce recall bias, yet short enough to ensure that the patient's condition remains stable. Such design considerations are addressed in the GRRAS guidelines [3].

This paper focuses on binary scales. In scenarios with more than two categories, the proportion of agreement and the kappa coefficients generalize easily. The proportion of specific agreement can be computed by selecting one category of interest and grouping all other categories together. When the scale is ordinal, other agreement measures, considering some distance metric between the ratings can be used. An overview of the statistical measures was recently published [60]. Furthermore, our focus was on intra‐ and inter‐rater studies. These study designs do not allow for the investigation of potential interaction effects between patients and observers. To explore such interactions, a more complex design is needed, specifically, one in which a sample of observers assesses a sample of patients on multiple occasions. This type of design enables the simultaneous study of both intra‐ and inter‐rater agreement. Developing appropriate agreement measures and statistical inference methods for such settings represents an important direction for future research.

## Conflicts of Interest

The authors declare no conflicts of interest.

## Supporting information




**Data S1.** Supporting Information.

## Data Availability

The data that support the findings of this study are openly available in simpleagree at https://github.com/svanbelle/simpleagree.

## Appendix A Standard Error of the Agreement Coefficients

The formulas for the large sample variance of the various agreement coefficients presented in Sections 4 and 5, derived by the delta method, are provided in this Appendix. The standard error is obtained by taking the square root of the variance. We denote the number of observers by R and the number of patients or objects by N.

### Proportion of Agreement

The large sample variance of the proportion of agreement is given by [53]

(A1)
$$\text{var}(p_{o})=\frac{1}{N^{2}}\left(\sum_{i=1}^{N}p_{oi}^{2}-Np_{o}^{2}\right)$$

where $p_{oi}$ is the proportion of agreement for patient i among the R observers (or the $R_{i}$ observers if the number of observers differs per patient, i=1,⋯,N). For the case of two observers, this reduces to the familiar binomial variance

(A2)
$$\text{var}(p_{o})=\frac{1}{N}p_{o}\left(1-p_{o}\right).$$

### Proportion of Specific Agreement

The large sample variance for the proportion of specific agreement is [53]

(A3)
$$\begin{array}{ll} & \text{var}(p_{pos})=\frac{1}{NB}\\ & \;\times \left[\left(2-(R-1)p_{pos}\right)\left(p_{pos}(1-(R-1)p_{pos})+\frac{12}{B}q_{3}\right)+\frac{24}{B}q_{4}\right] \end{array}$$

where $B=\sum_{p=1}^{R(R-1)/2}(p_{1.}^{\left(p\right)}+p_{.1}^{\left(p\right)})$ is the sum over all pairs p=1,⋯,P of the marginal proportions $p_{1.}^{\left(p\right)}$ and $p_{.1}^{\left(p\right)}$, while $q_{3}$ and $q_{4}$ are sums of the proportions of agreement on the positive test results in triplets and quartets of observers, respectively.

When there are only two observers, this reduces to [61]

(A4)
$$\text{var}(p_{pos})=\frac{1}{N(p_{1.}+p_{.1})}p_{pos}(1-p_{pos})(2-p_{pos}).$$

The large sample variance of the Jaccard index is derived from the above by

(A5)
$$\text{var}(p_{J})={\left(\frac{2}{{\left(2-p_{pos}\right)}^{2}}\right)}^{2}var(p_{pos}).$$

### Kappa Coefficients

Given that ${\hat{\kappa }}_{1}=2p_{o}-1$, we have

(A6)
$$\text{var}({\hat{\kappa }}_{1})=4\text{var}(p_{o}).$$

Under the chance definition 2 (Cohen's kappa), [62] showed that

(A7)
$$\begin{array}{cc} & \text{var}({\hat{\kappa }}_{2})\\ & =\frac{\sum_{i=1}^{N}{\left[(1-p_{e2})p_{oi}-2(1-p_{o})p_{e2i}\right]}^{2}⁄N-{\left(p_{o}p_{e2}-2p_{e2}+p_{o}\right)}^{2}}{N{\left(1-p_{e2}\right)}^{4}}\\ & \;-\frac{{\left(p_{o}p_{e2}-2p_{e2}+p_{o}\right)}^{2}}{N{\left(1-p_{e2}\right)}^{4}} \end{array}$$

where

$$p_{e2}=\frac{2}{R(R-1)}\overset{P}{\underset{p=1}{\sum }}(p_{1.}^{\left(p\right)}p_{.1}^{\left(p\right)}+p_{2.}^{\left(p\right)}p_{.2}^{\left(p\right)})$$

is the mean proportion of agreement expected by chance across all R(R−1)/2 distinct pairs of observers and

$$p_{e2i}=\frac{1}{R(R-1)}\overset{R}{\underset{r=1}{\sum }}\underset{r^{\prime }\neq r}{\sum }\;\overset{2}{\underset{j=1}{\sum }}p_{j(r)}y_{ij(r^{\prime })}$$

with $y_{ij(r^{\prime })}$ the rating of the second observer in the pair, and $p_{j(r)}$ the proportion of patients rated in category j by the first observer of the pair. For two observers, it reduces to

(A8)
$$\begin{array}{cc} \text{var}({\hat{\kappa }}_{2}) & =\frac{p_{o}(1-p_{o})}{N{\left(1-p_{e}\right)}^{2}}+\frac{2(p_{o}-1)(C_{1}-2p_{o}p_{e})}{N{\left(1-p_{e}\right)}^{3}}\\ & \;+\frac{{\left(p_{o}-1\right)}^{2}(C_{2}-4p_{e}^{2})}{N{\left(1-p_{e}\right)}^{4}} \end{array}$$

where

$$C_{1}=\overset{2}{\underset{j=1}{\sum }}p_{jj}(p_{j.}+p_{.j})\;\text{and}\;C_{2}=\overset{2}{\underset{j=1}{\sum }}\overset{2}{\underset{k=1}{\sum }}p_{jk}{\left(p_{.j}+p_{k.}\right)}^{2}.$$

Under chance definition 3, we have

(A9)
$$\begin{array}{cc} & \text{var}({\hat{\kappa }}_{3})=\frac{\sum_{i=1}^{N}{\left[(1-p_{e3})p_{oi}-2(1-p_{o})p_{e3i}\right]}^{2}⁄N-{\left(p_{o}p_{e3}-2p_{e3}+p_{o}\right)}^{2}}{N{\left(1-p_{e3}\right)}^{4}} \end{array}$$

where

$$\begin{array}{ll} p_{e3} & =\frac{2}{R(R-1)}\overset{P}{\underset{p=1}{\sum }}\left[{\left(\frac{p_{1.}^{\left(p\right)}+p_{.1}^{\left(p\right)}}{2}\right)}^{2}+{\left(\frac{p_{2.}^{\left(p\right)}+p_{.2}^{\left(p\right)}}{2}\right)}^{2}\right]=\frac{1}{N}\overset{N}{\underset{i=1}{\sum }}p_{e3i} \end{array}$$

and

$$p_{e3i}=\frac{1}{R}\overset{2}{\underset{j=1}{\sum }}m_{ij}p_{j},$$

with $m_{ij}$ is the number of observers classifying patient i in category j and $p_{j}=\sum_{i=1}^{N}m_{ij}/(NR)$ the overall proportion of patients classified in category j (j=1,2). If the number of observers differs between patients, R is replaced by $R_{i}$ in the formula of $p_{e3i}$. For two observers, the formula reduces to

(A10)
$$\begin{array}{lcr} \text{var}({\hat{\kappa }}_{3}) & =\frac{1}{N{\left(1-C_{3}\right)}^{2}}\left\{\sum_{j=1}^{2}p_{jj}[1-4{\bar{p}}_{j}(1-{\hat{\kappa }}_{3})]\right. & \\ & \;+{\left(1-{\hat{\kappa }}_{3}\right)}^{2}\sum_{j=1}^{2}\sum_{k=1}^{2}p_{jk}{\left({\bar{p}}_{j}+{\bar{p}}_{k}\right)}^{2} & \\ & \;\left.-{\left[{\hat{\kappa }}_{3}-C_{3}(1-{\hat{\kappa }}_{3})\right]}^{2}\right\} & \end{array}$$

where $p_{jk}=n_{jk}/N,j,k=1,2$, $p_{i.}=n_{i.}/N$, $p_{.j}=n_{.j}/N$, ${\bar{p}}_{j}=(p_{j.}+p_{.j})/2$ and $C_{3}={\bar{p}}_{1}+{\bar{p}}_{2}$.

For kappa coefficients, confidence intervals based on the Fisher‐Z transformation often have better statistical properties, especially when kappa is near 1. In that case, we first find the lower and upper limits on the transformed scale (L.KF and U.KF, respectively) and then transform these limits back. The transformed estimate is

$${\hat{\kappa }}_{F}=\frac{1}{2}\ln\left(\frac{1+\hat{\kappa }}{1-\hat{\kappa }}\right)$$

with standard error

$$\text{SE}({\hat{\kappa }}_{F})=\sqrt{\frac{(\text{SE}{\left(\hat{\kappa })\right)}^{2}}{{\left(1-{\hat{\kappa }}^{2}\right)}^{2}}}.$$

A Wald confidence interval on the transformed scale is then ${\hat{\kappa }}_{F}\pm Q_{Z}(1-\alpha /2)SE({\hat{\kappa }}_{F})$, which can be back‐transformed to the original scale using for the lower limit

$$L.K=\frac{\exp(2L.KF)-1}{\exp(2L.KF)+1}.$$

and similarly for the upper limit with U.KF replacing *L.KF*.

## Appendix B Sample Size Formulas

### Width of the Confidence Interval Approach

Suppose that for a fixed number of observers, we want to determine the minimum number of patients needed to reach a pre‐specified agreement value with a desired confidence interval width smaller or equal to *w*.

#### Two Observers

Denote the proportion of positive test results for observers 1 and 2 by $p_{1.}$ and $p_{.1}$, respectively. The minimum number of patients, N, needed to achieve a confidence interval width w around a planned agreement value is given by

(B1)
$$N=4Q_{z(1-\alpha /2)}^{2}\frac{y}{w^{2}}$$

where the form of y depends on the statistical measure.

For the proportion of agreement, we have

$$y=p_{o}(1-p_{o}).$$

For the proportion of positive agreement, we have

$$y=\frac{p_{pos}(2-p_{pos})(1-p_{pos})}{\left(p_{1.}+p_{.1}\right)}.$$

For the Jaccard coefficient, we have

$$y={\left(\frac{2}{{\left(2-p_{pos}\right)}^{2}}\right)}^{2}\frac{p_{pos}(2-p_{pos})(1-p_{pos})}{\left(p_{1.}+p_{.1}\right)}.$$

For the chance‐corrected agreement coefficient, we have

$$\begin{array}{llll} y & =\frac{p_{o}(1-p_{o})}{{\left(1-p_{e}\right)}^{2}}\; & +\frac{2(p_{o}-1)(C_{1}-2p_{o}p_{e})}{{\left(1-p_{e}\right)}^{3}} & +\frac{{\left(p_{o}-1\right)}^{2}(C_{2}-4p_{e}^{2})}{{\left(1-p_{e}\right)}^{4}} \end{array}$$

under chance definition 2 (Cohen's kappa coefficient).

On the other hand, under chance definition 3,

$$\begin{array}{cc} y & =\frac{1}{{\left(1-C_{3}\right)}^{2}}\left\{\overset{2}{\underset{j=1}{\sum }}p_{jj}[1-4{\bar{p}}_{j}(1-{\hat{\kappa }}_{3})]\right.\\ & \;+{\left(1-{\hat{\kappa }}_{3}\right)}^{2}\overset{2}{\underset{j=1}{\sum }}\overset{2}{\underset{k=1}{\sum }}p_{jk}{\left({\bar{p}}_{j}+{\bar{p}}_{k}\right)}^{2}\\ & \;\left.-{\left[{\hat{\kappa }}_{3}-C_{3}(1-{\hat{\kappa }}_{3})\right]}^{2}\right\} \end{array}$$

for the intraclass kappa coefficient.

For the proportion of agreement, the statistical properties of the confidence interval can be improved by using a continuity correction in the case of two observers. The minimum number of patients or objects in that case is equal to

(B2)
$$\begin{array}{l} N=\frac{\begin{array}{l} \left(2Q_{z(1-\alpha /2)}^{2}p_{o}(1-p_{o})+w+2Q_{z(1-\alpha /2)}\sqrt{Q_{z(1-\alpha /2)}^{2}p_{o}^{2}{\left(1-p_{o}\right)}^{2}+w}\right) \end{array}}{w^{2}}. \end{array}$$

#### More Than Two Observers

For more than two observers, the minimum number patients needed to achieve a width w around a planned proportion of agreement is given by Equation (B1) with

$$\begin{array}{l} y=\left(\overset{R}{\underset{r=0}{\sum }}s_{r}{\left(\frac{r(r-1)+(R-r)(R-r-1)}{R(R-1)}\right)}^{2}-p_{o}^{2}\right) \end{array}$$

for the proportion of agreement and

$$\begin{array}{ll} & y=\frac{1}{B}\left[\left(2-(R-1)p_{pos}\right)\left(p_{pos}(1-(R-1)p_{pos})+\frac{12}{B}q_{3}\right)+\frac{24}{B}q_{4}\right] \end{array}$$

for the proportion of positive agreement where $q_{3}$ and $q_{4}$ were previously defined. For the Jaccard coefficient, the y for positive agreement has to be multiplied by ${\left(\frac{2}{{\left(2-p_{pos}\right)}^{2}}\right)}^{2}$. For kappa coefficients, under chance definition 2, we have

$$\begin{array}{lcr} N & =\frac{1}{{\left(1-p_{e2}\right)}^{4}w^{2}}\left(-2Q_{z(1-\alpha /2)}^{2}{\left(p_{o}p_{e2}-2p_{e2}+p_{o}\right)}^{2}\right. & \\ & \;+\left.2\sqrt{\begin{array}{l} Q_{z(1-\alpha /2)}^{4}{\left(p_{o}p_{e2}-2p_{e2}+p_{o}\right)}^{4}+w^{2}{\left(1-p_{e2}\right)}^{4}\\ \overset{N}{\underset{i=1}{\sum }}{\left[(1-p_{e2})p_{oi}-2(1-p_{o})p_{e2i}\right]}^{2} \end{array}}\right) & \end{array}$$

and under chance definition 3, we have

$$\begin{array}{lcr} N & =\frac{1}{{\left(1-p_{e1}\right)}^{4}w^{2}}\left(-2Q_{z(1-\alpha /2)}^{2}{\left(p_{o}p_{e1}-2p_{e1}+p_{o}\right)}^{2}\right. & \\ & \;+\left.2\sqrt{\begin{array}{l} Q_{z(1-\alpha /2)}^{4}{\left(p_{o}p_{e1}-2p_{e1}+p_{o}\right)}^{4}+w^{2}{\left(1-p_{e1}\right)}^{4}\\ \overset{N}{\underset{i=1}{\sum }}{\left[(1-p_{e1})p_{oi}-2(1-p_{o})p_{e1i}\right]}^{2} \end{array}}\right) & \end{array}$$

When more than two observers are involved, additional information beyond the expected agreement levels and the expected proportion of positive test results is required to determine the minimum number of patients needed. For example, when considering the proportion of agreement, one must know the distribution of positive test results across r=0,…,R observers. Similarly, for the proportion of positive agreement, the quantities $q_{3}$ and $q_{4}$ are also necessary. If these values are unknown, we propose to simulate a large number of possible classification tables based on the planned agreement value and proportion of positive test results. From these simulations, the additional quantities are estimated for each table. Using these estimates, the minimal number of patients required is computed using the formulas presented in this section. Researchers can then select, for a given number of observers, a minimum number of patients or objects that covers a chosen proportion of the simulated classification tables (e.g., 90%). In the CTG example, a coverage of 100% of the classification tables was chosen, representing the worst‐case scenario. This simulation‐based procedure is implemented in the shiny app “simpleagree” (link: https://svanbelle.shinyapps.io/simpleagree/). Explanations on how to use the app are given as Supporting Information.

### Testing Approach

In the testing approach, suppose that we would like to test the hypotheses

$$H_{0}:\rho =\rho_{0}\;\text{versus}\;H_{1}:\rho >\rho_{0}\;\text{(i.e.,}\;\rho =\rho_{A})$$

with a power equal to 1−β and a type‐one error equal to α. The Greek letter ρ represents either the probability of (positive) agreement or the population value of a kappa coefficient.

The minimum number of persons needed to test these statistical hypotheses with a power of at least 1−β is given by

$$N\geq {\left(\frac{z_{1-\alpha }y(\hat{\rho }|\rho =\rho_{0})+z_{1-\beta }y(\hat{\rho }|\rho =\rho_{A})}{\rho_{0}-\rho_{A}}\right)}^{2}$$

where y is given in the previous section for the various coefficients.

Except when there are two observers, no analytical solution can be found. So, here too, the same simulation procedure is performed [53].
