# Supplementary material for: Measuring Agreement in Diagnostics: A Practical Guide for Researchers
Source: Stat Med. 2025 Oct 16;44(23-24):e70299. doi: 10.1002/sim.70299 (PMC12531475; doi:10.1002/sim.70299)
Supplement: Supplementary file 1 — Data S1. Supporting Information. [file SIM-44-0-s001.zip › Manualv200825.pdf]

Shiny application simpleagree
Version 1.0 (20.08.2025)
Contact: sophie.vanbelle@maastrichtuniversity.nl

Access to the app: https://svanbelle.shinyapps.io/simpleagree/

1. Instructions to upload your dataset

Please follow the guidelines below to ensure your dataset is correctly formatted and accepted by the system:

1. **File Format**: Upload your dataset as a CSV (comma-separated values) file. The file extension should be .csv.
2. **Data Encoding**:
   - Positive test results must be coded as 1.
   - Negative test results must be coded as 0.
3. **Data Structure**:
   - Each row should represent one patient or object.
   - Each column should represent one replicate, such as a time point or an observer.
4. **Allowed Delimiters**: The CSV file can use any of the following as column separators: comma, semicolon, tab character
5. **Missing Values**: Ensure your dataset does not contain any missing values. All fields must be filled with either 0 or 1.

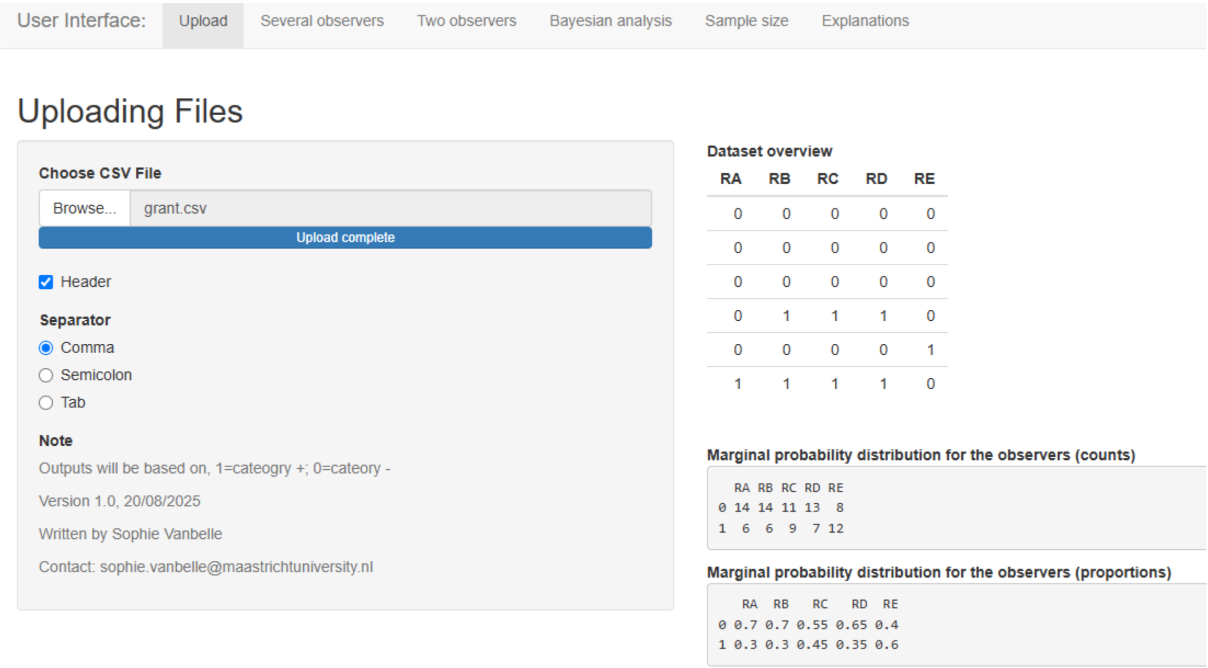

Once your dataset is uploaded successfully, the system will automatically generate summary statistics for each observer:

- The number of positive and negative test results (counts) will be displayed.
- The corresponding proportions of positive and negative results will also be calculated and shown.

These summaries help you quickly review the distribution of test results across all observers.

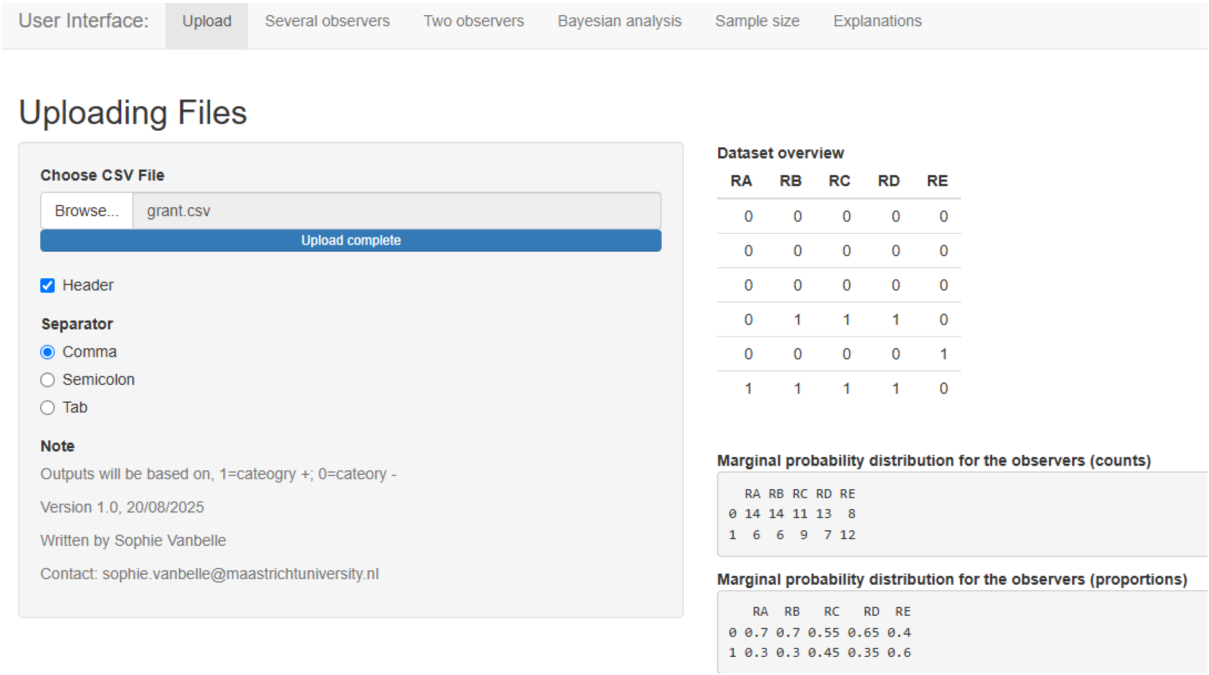

You can remark that observer E (RE) provided a higher proportion of positive test results (60%) compared to the other four observers, whose proportions ranged from 30% to 45%.

2. Main Menu Options

The main menu provides several features for analyzing your data:

1. **Agreement Between Multiple Observers**: Select the "Several Observers" tab to analyze the agreement between multiple observers.
2. **Agreement Between Two Observers**: Choose the "Two Observers" tab if you want to compute the agreement between two specific observers.

All statistical analyses are performed within the frequentist framework, which includes the calculation of 95% confidence intervals.

3. **Bayesian Analysis**: If you prefer to work within the Bayesian framework, you can do so by selecting the "Bayesian Analysis" tab. Please note that this feature is not covered in the manuscript.
4. **Sample Size Calculations**: Use the "Sample Size" tab to calculate the required sample size for your study.
5. **Explanations**: For detailed information on the methods and calculations used, visit the "Explanations" tab.

3. Study the agreement between several observers

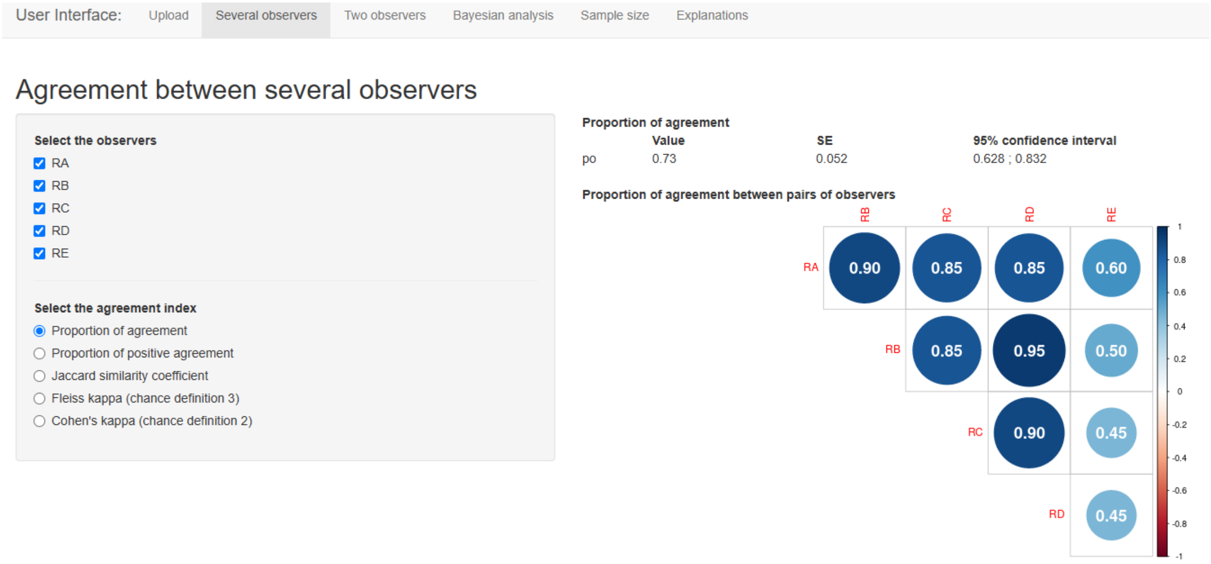

In the left tab, you can choose which observers to include in the statistical analysis and which statistic to compute. The available statistical measures include: the proportion of agreement, the proportion of positive agreement, Jaccard similarity coefficient, Fleiss kappa (corresponding to chance definition 3) or Cohen's kappa (corresponding to chance definition 2).

For example, in the screenshot above, we request the proportion of agreement for observers A to E. The estimate is displayed on the right of the screen (po=0.73) with its standard error (SE=0.052) and 95% Wald confidence interval (0.628; 0.832).

Additionally, the statistical measure is shown in a correlogram for all pairs of observers. In a correlogram, the color and the size of the circles are proportional to the agreement levels. Larger and darker circles indicate higher agreement while smaller and lighter circles indicate lower agreement.

In the correlogram, we can observe that pairs involving observer E show lower agreement compared to the other pairs. This may be due to observer E rating more patients as positive than the other observers.

4. Study the agreement between two observers

In the "Two Observers" tab, you can gain deeper insight into the agreement between a specific pair of observers. To do so:

1. Select the two observers you want to compare from the left menu (e.g., RA and RE).
2. Choose the statistical measure you'd like to compute (e.g., proportion of agreement).

This feature allows for a focused analysis on the agreement between just those two observers, providing more detailed results.

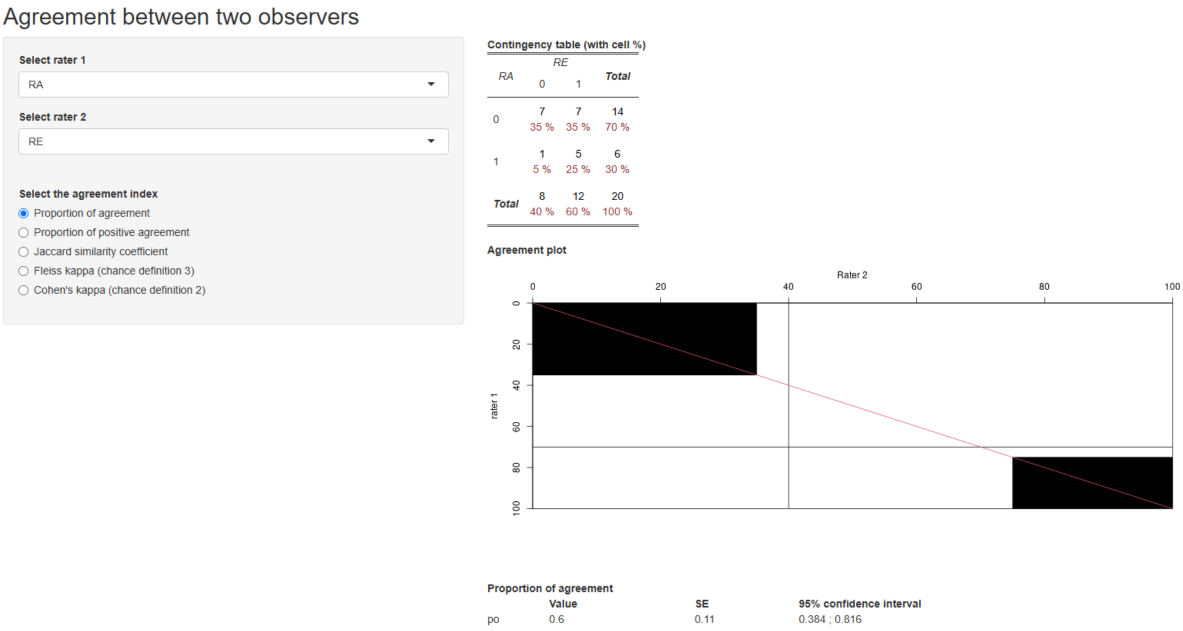

In the right panel, you'll see the classification table, which presents the data in terms of both counts and percentages. Additionally, an agreement plot is provided, offering a visual summary of the data. For more details on how to interpret this plot, please refer to the "Explanations" tab.

For example, you may observe that among the 14 negative test results of RA, 7 are classified as negative and 7 as positive by RE.

5. Bayesian statistical analysis

Bayesian statistical inference is based on the paper:

Vanbelle, S. (2024). Statistical inference for agreement between multiple raters on a binary scale. *British Journal of Mathematical and Statistical Psychology*, 77, 245–260. https://doi.org/10.1111/bmsp.12333

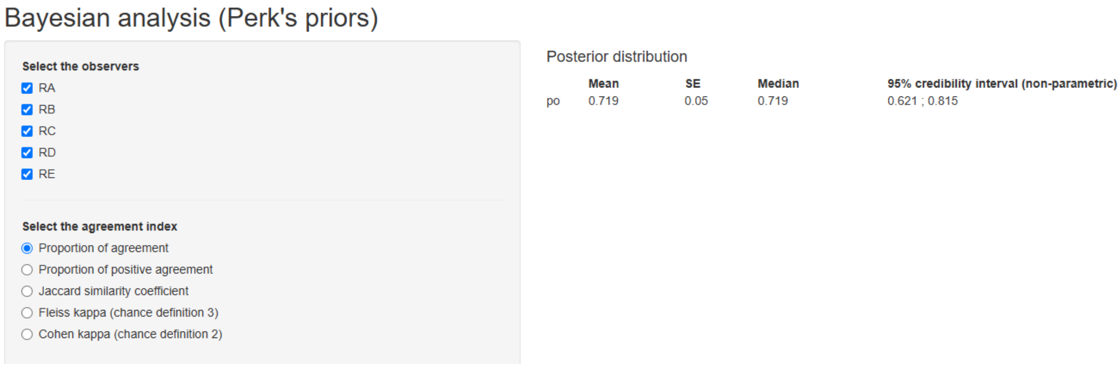

The posterior distribution of the different agreement coefficients is summarized in the right panel, including the posterior mean, standard error, median and 95% non-parametric percentile interval.

In the example, the 95% credibility interval is close to the 95% confidence interval.

6. Sample size calculation

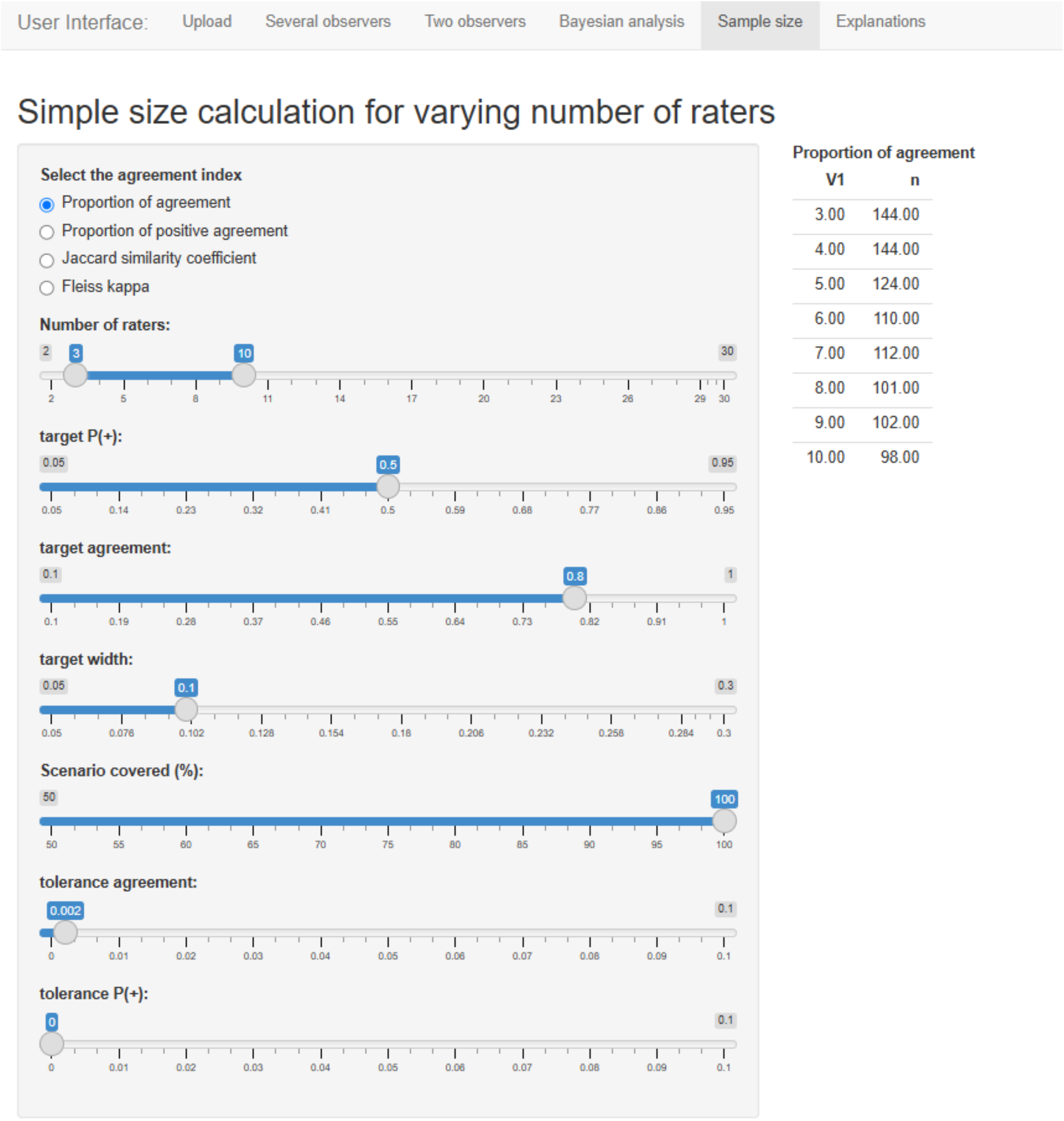

The sample size calculation is based on the width of the confidence interval approach. To perform the calculation, you'll need to select several parameters from the left panel:

1) The statistical measure of interest under "select the agreement index"
2) A range for the number of raters (between 2 and 30): "Number of raters"
3) The expected proportion of positive test results (between 0.05 and 0.95): "target P(+)"
4) The expected width of the confidence interval (between 0.05 and 0.3): "target width"
5) The % of scenario covered (between 50 and 100%): "Scenario covered (%)"

Sometimes, it may not be possible to compute the sample size with the values provided in points 2 to 4. If this happens, an error message will appear in the right panel in red.

To resolve this, you can adjust the tolerance for the expected agreement and the expected proportion of positive test results:

- Tolerance Agreement: Between 0 and 0.1.
- Tolerance P(+): Between 0 and 0.1.

Even a small tolerance can allow the calculation to proceed.

For example, with 6 observers, a minimum of 110 patients is needed to achieve a proportion of agreement of 0.8, a target confidence interval width of 0.1, and 100% scenario coverage.
